# Supplementary material for: Distinct Clusters of Testosterone Levels, Symptoms, and Serum Trace Elements in Young Men: A Cross-Sectional Analysis
Source: Nutrients. 2025 Feb 28;17(5):867. doi: 10.3390/nu17050867 (PMC11901754; doi:10.3390/nu17050867)
Supplement: Supplementary file 1 [file nutrients-17-00867-s001.zip › nutrients-3470529-supplementary.pdf]

Distinct Clusters of Testosterone Levels, Symptoms, and Serum Trace Elements in Young Men: A Cross-Sectional Analysis

Takazo Tanaka, Kosuke Kojo, Takahiro Suetomi, Yoshiyuki Nagumo, Haruhiko Midorikawa, Takaaki Matsuda, Ayumi Nakazono, Takuya Shimizu, Shunsuke Fujimoto, Atsushi Ikeda, Shuya Kandori, Hiromitsu Negoro, Tatsuya Takayama and Hiroyuki Nishiyama

Additional File S1: Supplementary materials

Page 2. Supplementary Table S1. Summary of the results of semen analysis

Page 3. Supplementary Table S2. Summary of the results of explanatory variables.

Page 4. Supplementary Figure S1. Eligibility criteria.

Page 5. Supplementary Figure S2. Heatmap of the correlation matrix of explanatory variables

Page 6. Supplementary Figure S3. Elbow method for determining the number of clusters

Page 7. Supplementary Figure S4. Silhouette analysis for k-means clustering with k = 2 to 5

Table S1: Summary of the results of semen analysis

	Median (IQR)	LLR	Below LLR (%)
Semen Volume (mL)	3.50 (2.60–4.50)	1.4	5
Sperm Concentration (×10^6/mL)	38.80 (9.40–95.90)	16	32
Sperm Motility (%)	52.30 (34.60–67.60)	42	34
Total sperm count (×10^6)	138.00 (34.85–337.50)	39	28
(at least one below LLR)			51

IQR, interquartile range; LLR, lower limit of reference intervals (cut-off values).

Table S2: Summary of the results of explanatory variables

	Median (IQR)	LLR	Below LLR (%)
Testosterone (ng/mL)	4.45 (3.57–5.47)	2.5	6
Free testosterone (pg/mL)	10.60 (8.50–12.90)	7.5	15
(At least one below LLR)			17
(Both below LLR)			4
AMS (points)	28.00 (23.00–33.00)		
17–26 (no/little complaints)	42%		
27–36 (mild complaints)	41%		
37–49 (moderate complaints)	16%		
50–85 (severe complaints)	1.3%		
Somatic subdomain (points)	12.00 (9.00–14.00)		
Psychological subdomain (points)	6.00 (5.00–8.00)		
Sexual subdomain (points)	9.00 (7.00–12.00)		
EHS (points)	3.44 (0.05)[1]		
Grade 1	2.2%		
Grade 2	4.0%		
Grade 3	42%		
Grade 4	52%		

IQR, interquartile range; LLR, lower limit of reference intervals (cut-off values); AMS, Aging Male's Symptom (Scale); EHS, Erection Hardness Score.

1) mean (standard error)

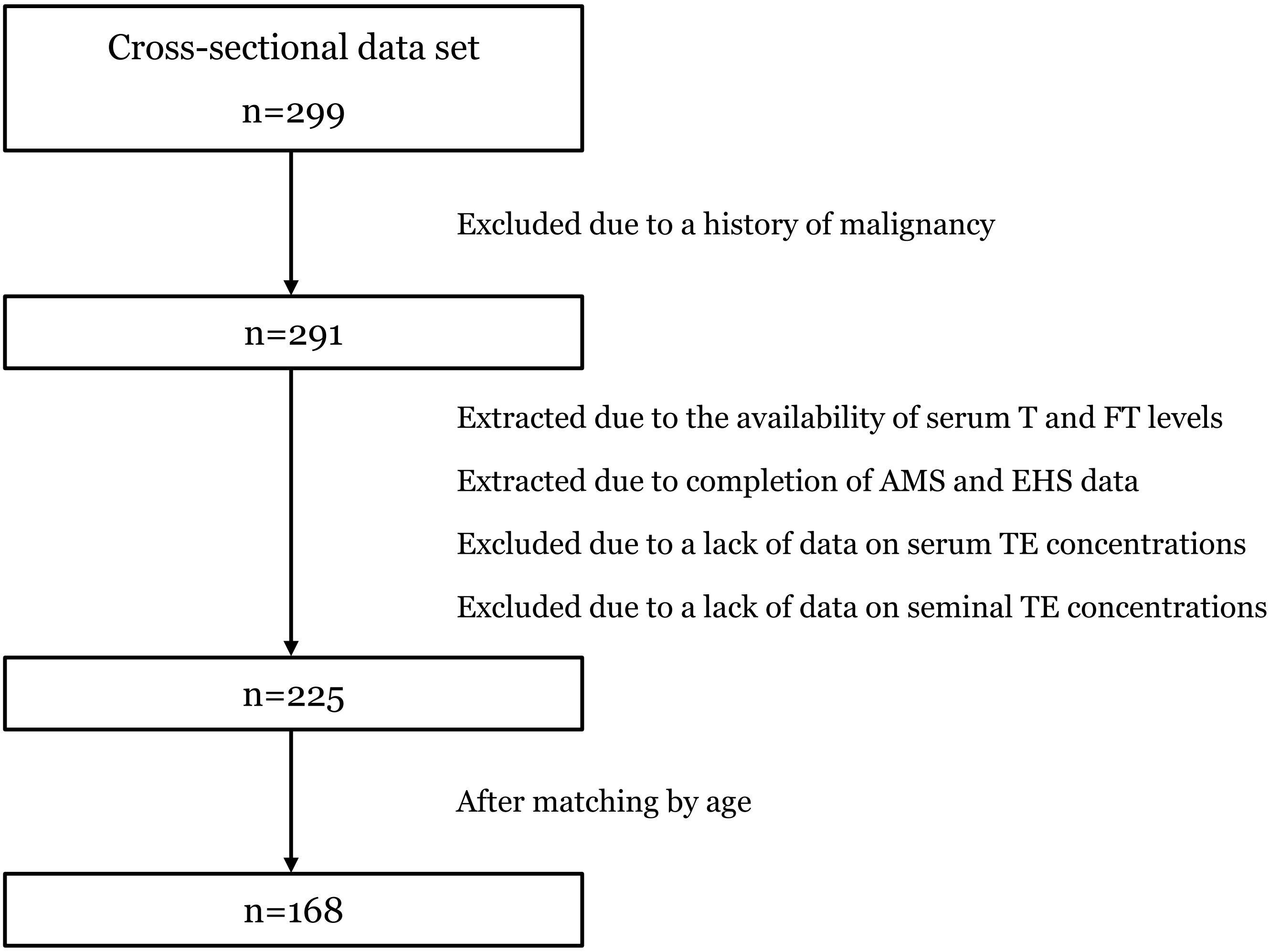

Figure S1: Eligibility criteria

T, total testosterone; FT, free testosterone; AMS, Aging Male's Symptom (Scale); EHS, Erection Hardness Score; TE, trace element

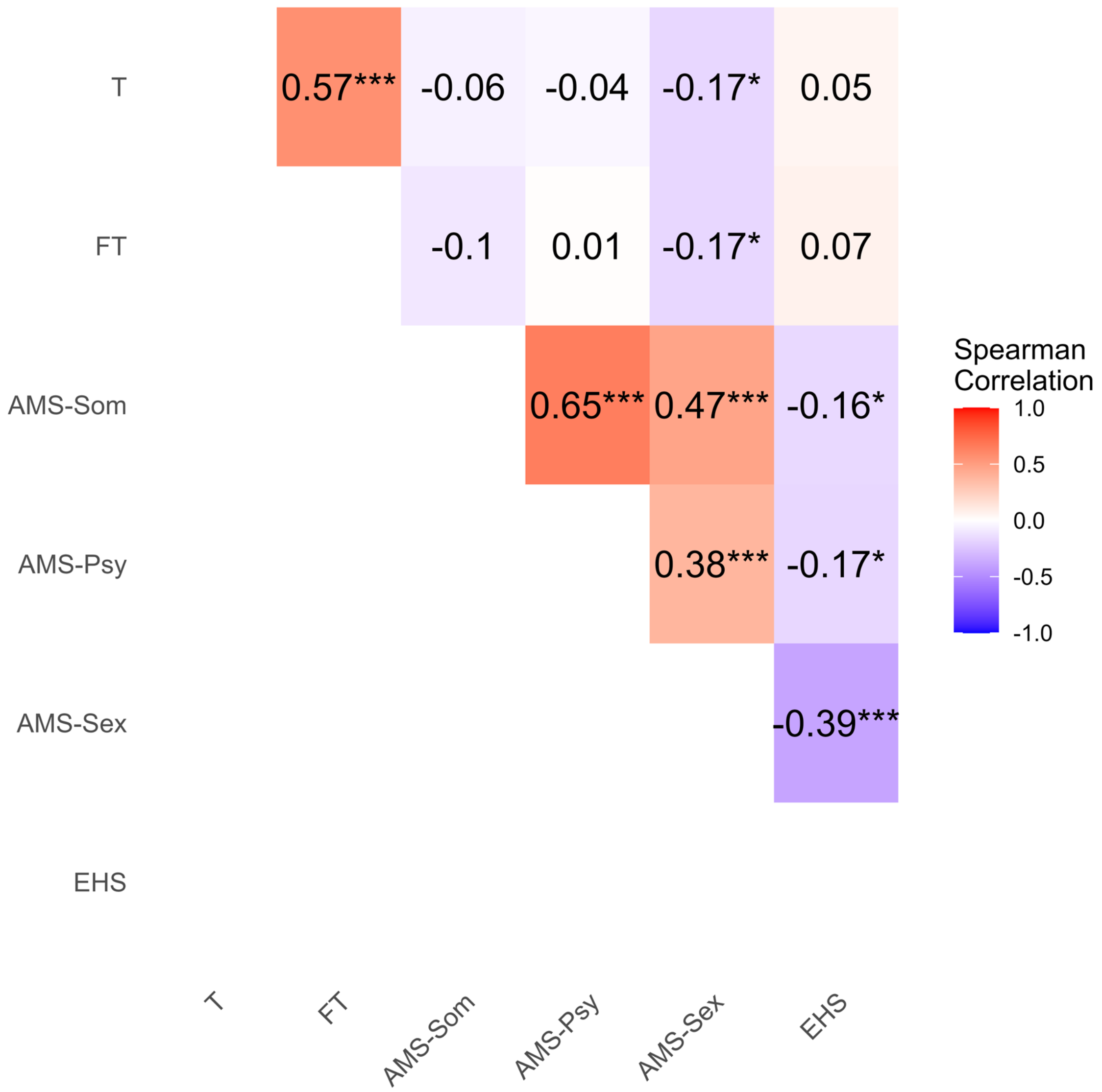

Figure S2: Heatmap of the correlation matrix of explanatory variables

T, total testosterone, FT, free testosterone; AMS; Aging Male's Symptom (Scale); AMS-Som, somatic subdomain of AMS; AMS-Psy, a psychological subdomain of AMS; AMS-Sex, sexual subdomain of AMS; EHS, Erection Hardness Score. The values represent Spearman's rank correlation coefficients (Spearman's "rho"). * indicates $p < 0.05$, and *** indicates $p < 0.001$.

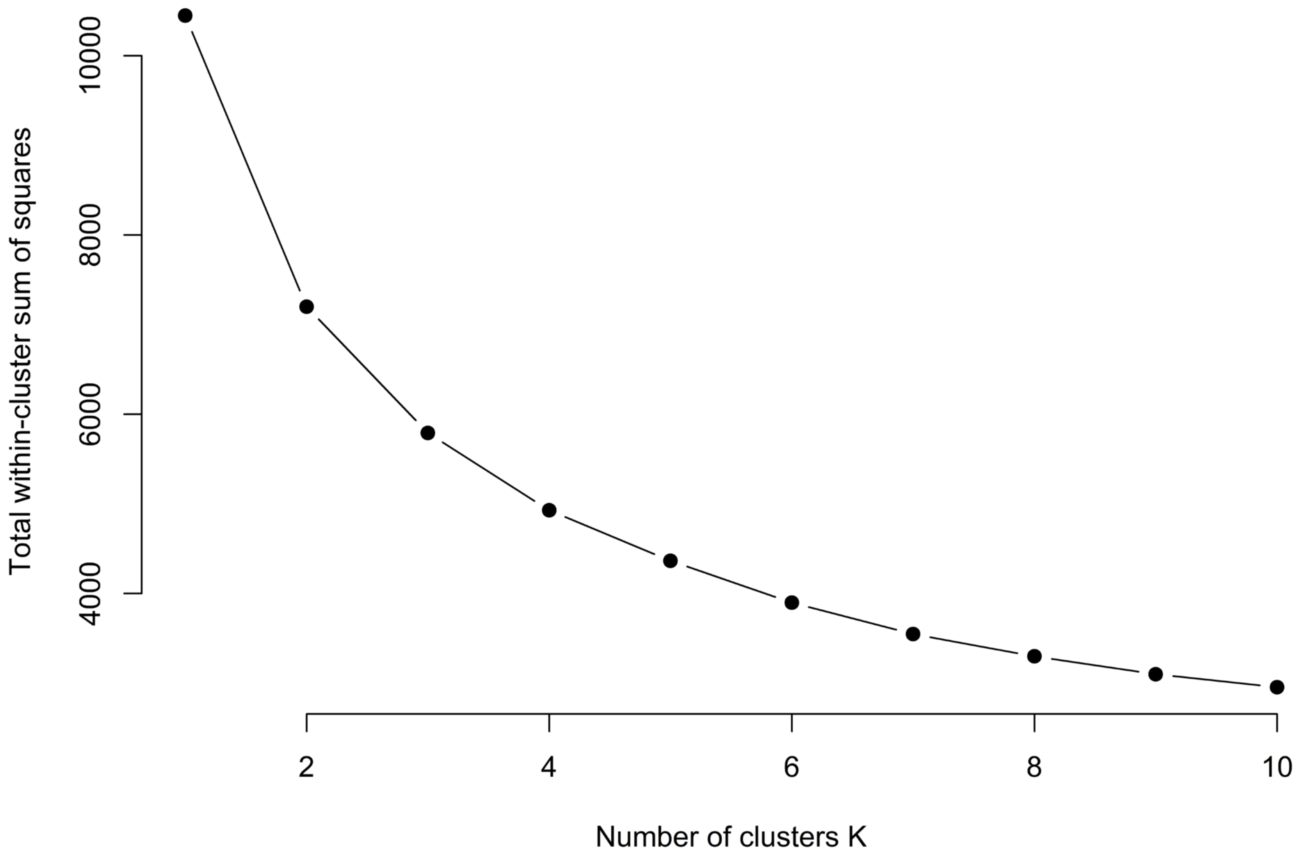

Figure S3: Elbow method for determining the number of clusters

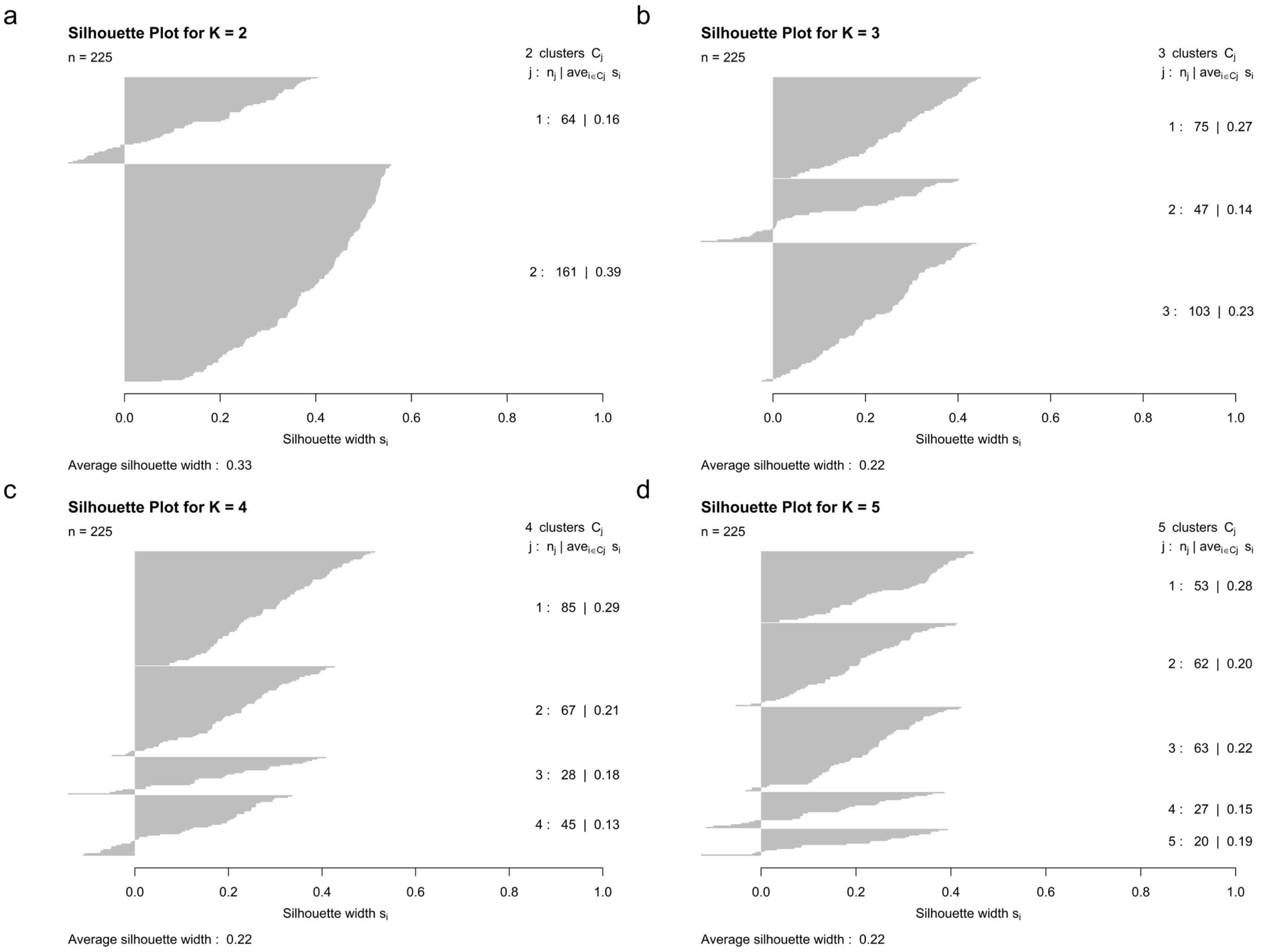

Figure S4: Silhouette analysis for k-means clustering with k = 2 to 5

Silhouette plots for k-means clustering configurations with k = 2 (a), k = 3 (b), k = 4 (c), and k = 5 (d). Each plot displays the silhouette coefficients of the individual data points, with cluster averages indicated by dashed lines. The height of each bar corresponds to the silhouette width of a point, reflecting its cohesion within the assigned cluster and its separation from other clusters. Moderate or low average silhouette values across all tested k values highlight the continuous nature of the data and the absence of distinct boundaries among clusters.
